# The Role of Positive Psychological Factors in the Association between Pain Intensity and Pain Interference in Individuals with Chronic Musculoskeletal Pain: A Cross-Sectional Study

**DOI:** 10.3390/jcm9103252

**Published:** 2020-10-12

**Authors:** Javier Martinez-Calderon, Mar Flores-Cortes, Susana Clavero-Cano, Jose Miguel Morales-Asencio, Mark P. Jensen, Antonio Rondon-Ramos, Juan Luis Diaz-Cerrillo, Gina Rocío Ariza-Hurtado, Alejandro Luque-Suarez

**Affiliations:** 1 Departamento de Fisioterapia, Facultad de Ciencias de la Salud, Universidad de Málaga, 29071 Málaga, Spain; calderonjmc@uma.es (J.M.-C.); marflores@uma.es (M.F.-C.); antonio.rondon.sspa@juntadeandalucia.es (A.R.-R.); aluques@uma.es (A.L.-S.); 2 Instituto de Investigación Biomédica de Málaga (IBIMA), 29071 Malaga, Spain; jmmasen@uma.es; 3 Servicio Andaluz de Salud, Distrito de Atención Primaria Costa del Sol, U.G.C. Las Albarizas, 29600 Marbella, Málaga, Spain; 4 Department of Nursing, Faculty of Health Sciences, Universidad de Malaga, 29071 Malaga, Spain; 5 Department of Rehabilitation Medicine, University of Washington, Seattle, WA 98104, USA; mjensen@uw.edu; 6 Servicio Andaluz de Salud, Distrito de Atención Primaria Costa del Sol, U.G.C. Las Lagunas, 29650 Mijas, Málaga, Spain; 7 Servicio Andaluz de Salud, Distrito de Atención Primaria Costa del Sol, U.G.C. La Carihuela, 29620 Torremolinos, Málaga, Spain; juanl.diaz.cerrillo.sspa@juntadeandalucia.es; 8 Servicio Andaluz de Salud, Distrito de Atención Primaria Costa del Sol, U.G.C. San Pedro de Alcántara, 29670 Marbella, Málaga, Spain; ginar.ariza.hurtado.sspa@juntadeandalucia.es

**Keywords:** chronic pain, cognition, cross-sectional studies, musculoskeletal pain, optimism, pain, self-efficacy

## Abstract

This study aimed to test the cross-sectional mediating and moderating role that positive psychological factors play in the association between pain intensity and pain interference in individuals with chronic musculoskeletal pain. A descriptive cross-sectional study using mediation analyses was conducted, including 186 individuals with chronic musculoskeletal pain. We conducted cross-sectional mediation and moderation analyses to determine whether the positive psychological factors mediated or moderated the association between pain intensity and pain interference. Pain acceptance, pain self-efficacy, and optimism were all significantly and weakly related to pain interference when controlling for pain intensity. Pain self-efficacy and pain acceptance partially mediated the association between pain intensity and pain interference. On the other hand, the multiple mediation model did not show significant effects. The three positive psychological factors were not found to significantly moderate the association between pain intensity and pain interference. The findings suggest that in chronic musculoskeletal pain patients, the treatments may focus on [i] what they are capable of doing to manage the pain (i.e., pain self-efficacy) and [ii] being better able to accept the pain as pain waxes and wanes might be also particularly helpful. However, these results must be tested in longitudinal studies before drawing any causal conclusion.

## 1. Introduction

One in three people lives with chronic musculoskeletal pain [1], which is one of the leading causes of years lived with disability [2]. Chronic musculoskeletal pain is known to be associated with a substantial burden in terms of greater lack of work productivity [3], medication overuse [4], healthcare expenditures [5], loneliness [6], mental health decline [7], suicidal behaviors [8], and premature mortality [7].

Psychological factors can influence how people process and manage their pain [9,10,11]. These factors can be part of an individual’s day-to-day life (e.g., stress [12] or social support [13]) or emerge in response to pain (e.g., fear of pain [14] or pain acceptance [15]). Although much of the focus of research on psychological factors and chronic pain has focused on maladaptive responses—in particular catastrophizing [16]—positive psychological factors could also potentially play an important role in the adjustment to persistent pain [17,18]. Recently, some pain adaptation paradigms have emerged aiming to explain why some people respond better than others to the chronic pain experience [17,18]. For example, Sturgeon and Zautra propose that resilience resources and mechanisms such as optimism, purpose in life, and acceptance can determine how people with chronic pain can adapt to that pain [17]. Furthermore, McCracken and Morley suggest that psychological flexibility, an integral concept based on different positive psychological constructs such as cognitive defusion and acceptance, is a key factor to persist and change negative behaviors in response to pain [18]. These factors have been shown to foster positive health behaviors, physical activity participation, social integration, and adaptive management of pain-related distress [11,19,20,21].

Pain acceptance, pain self-efficacy, and optimism are probably the positive psychological factors that have received more empirical attention in the context of chronic pain [19,22], and they have been shown to play a role in the adjustment of that pain [19,22,23]. Pain acceptance is defined as a general willingness to experience pain and engagement in activities despite that pain [18]. Pain self-efficacy is conceptualized as the ability to conduct a determined movement and/or activity and produce the desired outcome despite pain [24]. Optimism refers to the tendency to maintain positive expectations in spite of uncertainties about the future [25]. These factors are shown to be significantly associated with greater active coping skills, which may help people with chronic pain to respond to pain with more adaptive thoughts, optimistic self-statements, goal setting, and activity pacing behaviors [26,27,28]. Pain acceptance, pain self-efficacy, and optimism have also all been shown to be associated with less disability, pain, disease activity, distress, and medication use [19,20,29,30,31], as well as with greater positive affect, well-being, hope, and treatment adherence behaviors [30,31,32,33].

A number of researchers evaluated the potential cross-sectional mediator role that positive psychological factors play in the association between musculoskeletal pain intensity and a variety of pain-related outcomes [34,35]. For example, pain self-efficacy was shown to cross-sectionally mediate the association between pain intensity and depression in a sample of individuals with fibromyalgia [34]. Additionally, one facet of pain acceptance—activity engagement—was shown to cross-sectionally mediate the association between self-compassion and depressive symptoms in a sample of individuals with chronic musculoskeletal pain [36]. Similarly, pain self-efficacy and another facet of pain acceptance—pain willingness—were shown to cross-sectionally mediate the association between pain intensity and engagement in valued life activities in a sample of individuals with rheumatoid arthritis [35].

Another study used longitudinal data to evaluate the mediator role that self-efficacy beliefs play in the association between pain intensity and disability in individuals with chronic low back pain [37]. This study concluded that self-efficacy beliefs partially mediated the relationship between changes in pain and changes in disability over 12 months [37]. In samples of individuals with chronic pain, several studies have supported the conclusions that both self-efficacy beliefs [38] and pain acceptance [39,40,41,42] mediate the improvements in outcome produced by psychological and multidisciplinary pain treatments.

However, to our knowledge, no study has tested the mediating effects of multiple positive psychological factors such as pain acceptance, pain self-efficacy, and optimism in the association between pain intensity and pain interference in the same sample with chronic musculoskeletal pain. Such research would allow us to compare the relative importance of these three positive psychological factors as mediators.

In addition to possibly mediating the association between pain intensity and pain interference, positive psychological factors might also moderate the association between pain intensity and pain interference, such that those with higher levels of the positive psychological factors may evidence weaker associations between pain intensity and interference than those with lower levels of these factors. This result would occur if the positive psychological factors buffer the impact of pain intensity on pain interference, in the same way that positive social support is sometimes found to buffer the impact of stress on psychological and health outcomes [43]. However, to our knowledge, no researchers have evaluated the potential moderating effects that positive psychological factors play in the association between pain intensity and pain-related outcomes such as pain interference.

Given these considerations, the current study had three purposes. First, we sought to estimate the sizes of the direct associations between three positive psychological factors (i.e., pain acceptance, pain self-efficacy, and optimism) and pain interference in a sample of individuals with chronic musculoskeletal pain. Second, we sought to test whether these positive psychological factors cross-sectionally moderated the association between pain intensity and pain interference. Third, we sought to test the cross-sectional mediating roles of pain acceptance, pain self-efficacy, and optimism in the association between pain intensity and pain interference in the study sample. Given previous research in this area [34,35,36,37] and the theoretical pain frameworks that hypothesized the beneficial effects of positive psychological factors in the adjustment of chronic pain [17,18], we hypothesized [i] significant negative associations between measures of the three positive psychological factors and pain interference, [ii] significant cross-sectional mediation effects for the positive psychological factors in the association between pain intensity and pain interference, and [iii] significant cross-sectional buffering/moderation effects for the positive psychological factors, such that the association between pain intensity and pain interference would be weaker for those endorsing higher levels of the positive psychological factors. Although our study was cross-sectional and we cannot draw causal conclusions from mediation analyses of cross-sectional data, cross-sectional mediation analyses can still be performed as a way to develop hypotheses about possible causal associations that can then be tested with longitudinal designs.

## 2. Materials and Methods

### 2.1. Study Design

We conducted a cross-sectional study that followed the Declaration of Helsinki and the Strengthening the Reporting of Observational Studies in Epidemiology (STROBE) statement for observational studies [44]. We obtained the ethical board approval letter from the local Ethics Committee.

### 2.2. Participants and Setting

We recruited 186 people with chronic musculoskeletal pain from four primary care centers from September 2017 to December 2018. The recruitment was conducted by one physiotherapist at each center. Two research staff members with experience in the management of chronic musculoskeletal pain trained physiotherapists in a one-hour session. This session focused on clarifying eligibility criteria to facilitate physiotherapists the recruitment. Those participants who satisfied the eligibility criteria were invited to participate and provide written informed consent.

### 2.3. Eligibility Criteria

Participants were included if: [i] they were aged 18 or older; [ii] and presented a primary complaint of chronic musculoskeletal pain according to the multidimensional diagnostic criteria for chronic pain [45]. Inside of the broad concept of chronic musculoskeletal pain, specific pain conditions such as chronic myofascial pain, fibromyalgia, chronic widespread pain, gout, osteoarthritis, rheumatoid arthritis, and spondyloarthropathies were considered for inclusion. However, no rheumatic diseases were found among the participants. There were neither ethnicity nor gender restrictions. We excluded people with [i] a history of musculoskeletal trauma (e.g., fracture); [ii] postoperative musculoskeletal pain during the previous six months; [iii] musculoskeletal pain suspected to be originated from neurological (e.g., stroke), neoplastic (e.g., breast cancer) and/or referred pain (e.g., visceral referred pain) and; [iv] participants unable to provide written informed consent.

### 2.4. Sample Size Calculation

We determined a priori the sample size based on the results published by Costa and colleagues [37], who reported a determination coefficient of 0.34 for self-efficacy as a mediator between pain and disability and 0.10 for self-efficacy and disability. Assuming the weaker of these effects (i.e., determination coefficient of 0.10), a power of 90%, and an alpha of 0.05, we determined that 180 individuals would be needed to detect significant mediation effects.

### 2.5. Measures

We assessed pain intensity and pain interference using the Chronic Pain Grade Scale (CPGS) [46]. The pain intensity subscale is composed of three items where we estimated the characteristic pain intensity score as the sum of the 0–10 ratings of current, worst, and average pain [47]. This characteristic pain score could range from 0 to 30, with higher scores reflecting more pain intensity. The pain interference is also composed of three items where we estimated an overall pain interference score as the sum of the 0–10 ratings of the amount of difficulty performing daily, social, and work activities [47]. The pain interference score could range from 0 to 30, with higher scores reflecting greater pain interference. The Spanish version of the CPGS was used (internal consistency Cronbach’s α 0.87; test-retest reliability 0.81) [46]. The internal consistency Cronbach’s α in our sample was 0.93. This self-reported tool has been well-validated and its use is recommended in people with chronic pain [46,47,48].

We assessed pain self-efficacy using the 10-item Pain Self-Efficacy Questionnaire (PSEQ) [24]. Respondents rate each of the PSEQ items on a 0 (“not at all confident”) to 6 (“completely confident”) scale. The total score can range from 0 to 60, with higher scores reflecting more pain self-efficacy. In the original version of PSEQ, the internal consistency Cronbach’s α was 0.92 and the test-retest reliability was 0.73 [24]. The internal consistency Cronbach’s α in our sample was 0.93. We assessed pain acceptance using the 20-item Chronic Pain Acceptance Questionnaire (CPAQ) [49]. With the CPAQ, respondents rate each item on a 0 (“never true”) to 6 (“always true”) scale, and the total score is the sum of the responses. The total score can range from 0 to 120, with higher scores reflecting more pain acceptance [50]. The Spanish version of the CPAQ was used (internal consistency Cronbach’s α 0.83; test-retest reliability 0.83 [49]). The internal consistency Cronbach’s α in our sample was CPAQ activity engagement 0.85 and CPAQ pain willingness 0.78. Dispositional optimism was measured using the 10-item version of the Life Orientation Test-Revised (LOT-R) [51]. Respondents rate each item on a 0 (“strongly disagree”) to 4 (“strongly agree”) scale. The total scores are the sum of these ratings and can range from 0 to 40. Higher scores reflect more optimism. The Spanish version of the LOT-R was used [51] (internal consistency Cronbach’s α 0.73 [52]). The internal consistency Cronbach’s α in our sample was 0.63. We also assessed other demographic (i.e., age, gender, employment status, educational level, and presence of comorbidities) and pain history (i.e., pain duration, pain site, and primary treatment received) data.

### 2.6. Statistical Analysis

We evaluated all data for normality of distribution and values were all presented as mean ± standard deviation, unless otherwise stated. For descriptive purposes, we first estimated the magnitude of the univariate associations between the three positive psychological factors (pain acceptance, pain self-efficacy, and optimism) pain intensity, and pain interference. We calculated correlation coefficients before multivariable analysis. We interpreted these correlations as perfect (0.9 to 1); strong (0.7 to 0.9); moderate (0.4 to 0.6); weak (0.1 to 0.3), and zero (0) [53]. Before performing the planned regression analyses, we tested the variables for the assumptions for this analysis by examining the skew of their distributions (with plans to transform any variables that showed skewness >3.0) and evaluated potential multicollinearity by computing the variance inflation factors for each predictor (with plans to drop from the regression analysis any variable with a variance inflation factor >10 [54]. To determine the extent to which the positive psychological factors contributed independent variance to the prediction of pain interference when controlling for pain intensity, we regressed the measure of pain interference on pain intensity and the three positive psychological factors. Specifically, we entered the measure of characteristic pain intensity in the first step to control for the effects of this variable. Next, in step two, we entered the three psychological factors (pain acceptance, pain self-efficacy, and optimism) as a block. Finally, in step 3, we entered product terms representing the Pain Intensity X Pain Acceptance, Pain Intensity X Pain Self-Efficacy, and Pain Intensity X Optimism interaction effects. All these analyses were carried out by using the enter method.

Finally, we performed a cross-sectional mediation analysis to test the hypothesized mediation effects of the positive psychological factors in the association between pain intensity and pain interference. We conducted two cross-sectional mediation analyses (because the measure of optimism did not evidence a significant association with pain intensity, making it unlikely as a mediator of the association between pain intensity and pain interference). We established that a variable is a mediator when (1) the mediator (the psychological positive factor) showed a significant relationship with the independent variable (pain intensity) (path a); (2) the mediator was significantly associated with the outcome (pain interference) (path b); (3) the independent variable (pain intensity) was significantly related to the outcome (pain interference) (path c); and (4) the coefficient for the independent variable (pain intensity) and the outcome (pain interference) significantly decreased when the mediator (the psychological positive factor) was added to the model (path c′). These relations can be easily tested by using a cross-sectional mediation model [55]. Based on the assumptions of Hayes [56], we analyzed the indirect effect by using bootstrapping, with 10,000 resampling repetitions. The Sobel test was used to assess the significance of the cross-sectional mediation effect. We run the analysis with SPSS 25 statistical package (IBM SPSS Statistics for Windows, Version 25.0. Armonk, NY, USA: IBM Corp.) and PROCESS Procedure for SPSS Version 3.3 [57]. We used a *p*-value less than 0.05 to determine statistical significance. Missing values of the main variables were completely at random and were related to failures during the process of obtaining some questionnaires.

## 3. Results

### 3.1. Baseline Demographic and Clinical Characteristics

The sample descriptive information is presented in Table 1. As can be seen, we included 186 participants (71.5% female). The mean age of the sample was 52.7 years. Most of the participants reported symptoms for more than 12 months (87.1%). Low back pain was the most common pain location (42.6%). The mean of the dependent variable (GCPS-pain interference) was 17.5 (standard deviation (SD) 12.1).

### 3.2. Univariate Associations between Psychological Measures, Pain Intensity, and Pain Interference

The results of the descriptive univariate associations between the study variables are presented in Table 2. As can be seen, greater pain acceptance and pain self-efficacy were significantly associated with less pain intensity and pain interference. However, the measure of optimism evidenced a weak and non-significant association with both pain intensity and pain interference.

### 3.3. Linear Regression Model

The results of the regression analyses predicting pain interference are presented in Table 3. In step 2, pain acceptance (β = −0.28, 95% confidence interval (CI) = −0.26 to −0.09, *p* < 0.001), pain self-efficacy (β = −0.14, 95% CI = −0.23 to −0.01, *p* = 0.031), and optimism (β = 0.14, 95% CI = 0.07 to 0.76, *p* = 0.017) were significantly and weakly associated with pain interference. The predictive value of the model was large and significant (*R^2^ =* 0. 59, F (6141) = 34.33, *p* < 0.001). However, none of the positive psychological factors evidenced significant cross-sectional moderator effects in the association between pain intensity and pain interference (Pain Intensity X Pain Acceptance β = −0.06 *p* = 0.402; Pain Intensity X Pain Self-Efficacy: β = −0.04 *p* = 0.522; Pain Intensity X Optimism β = 0.01 *p* = 0.803). The regression model did not improve its determination coefficient with the addition of these predictors (R^2^ = 0.60).

### 3.4. Cross-Sectional Mediating Effects of Pain Self-Efficacy and Pain Acceptance

**Figure 1:**
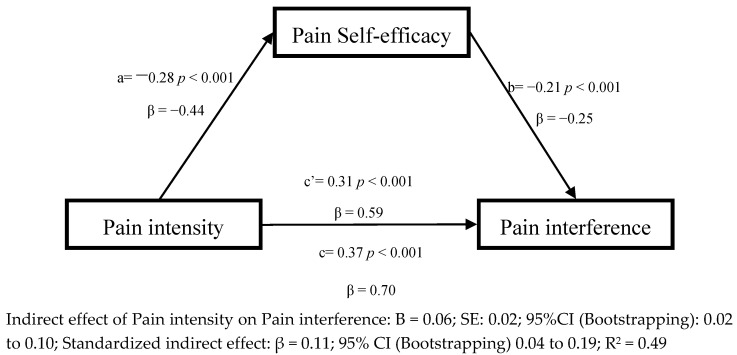
Linear regression results for the mediation model, adjusted by age and gender, with pain self-efficacy as the mediator between pain intensity and pain interference.

**Figure 2:**
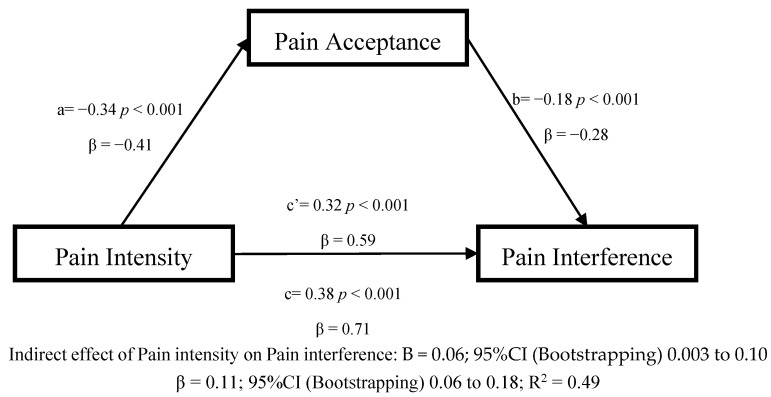
Linear regression results for the mediation model, adjusted by age and gender, with pain acceptance as the mediator between pain intensity and pain interference.

**Figure 3:**
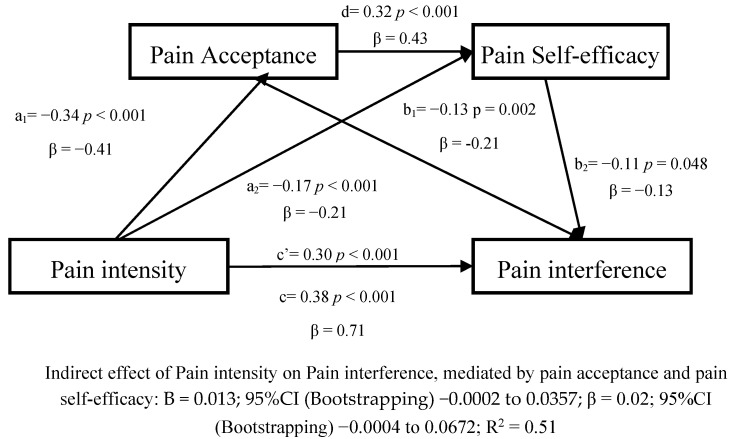
Linear regression results for the multiple mediation model, adjusted by age and gender, with pain acceptance and pain self-efficacy as the mediators between pain intensity and pain interference.

The results of the planned cross-sectional mediation analyses are presented in Figure 1, Figure 2 and Figure 3. As noted previously we only evaluated the cross-sectional mediation effects of pain self-efficacy and pain acceptance, adjusted by age and gender, because optimism evidenced only a weak and non-significant association with pain intensity in the univariate analyses. As can be seen, in the first model (Figure 1), and consistent with the regression analyses, greater pain intensity was associated with less pain self-efficacy (path a; β = −0.44, *p* < 0.001), and greater pain self-efficacy was associated with less pain interference (path b; β = −0.25, *p* < 0.001). Greater pain intensity was directly associated with more pain interference (path c′; β = 0.59, *p* < 0.001). The total effect of pain intensity on pain interference was also significant (path c; β = 0.70, *p* < 0.001). Finally, the indirect effect of pain intensity on pain interference showed a significant relation (β = 0.11; 95% CI (Bootstrapping) 0.04 to 0.19 Sobel test: S = 3.39; *p* < 0.0001), which suggests that pain self-efficacy partially mediated the association between pain intensity and pain interference in a cross-sectional manner.

In the second model evaluating the cross-sectional mediation effect of pain acceptance in the association between pain intensity and pain interference (Figure 2), greater pain intensity was associated significantly with less pain acceptance (path a; β = −0.41, *p* < 0.001), and less pain acceptance was associated with more pain interference (path b; β = −0.28, *p* < 0.001). Greater pain intensity was associated with more pain interference (path c; β = 0.71, *p* < 0.001). The indirect effect of pain intensity on pain interference showed a significant relation partially mediated by pain acceptance (β = 0.11, *p* < 0.001; 95%CI (Bootstrapping) 0.06 to 0.18; Sobel test: S = 3.57; *p* < 0.0001).

We evaluated a third model which introduced a multiple mediation model with pain acceptance and pain self-efficacy as mediators (Figure 3).

Concerning the preceding models, the association between pain intensity, pain acceptance, pain self-efficacy, and pain interference remained significant in the same. Furthermore, pain acceptance was significantly associated with pain self-efficacy (β = 0.43; *p* < 0.001). Nevertheless, the multiple mediation model did not obtain a significant effect (β = 0.02, 95%CI (Bootstrapping) −0.0004 to 0.0672; N.S.).

## 4. Discussion

Several systematic reviews have concluded that greater self-efficacy beliefs, including pain self-efficacy, are associated with and predict less pain intensity and disability in people with chronic pain [20,22]. Observational studies have found that greater pain acceptance is linked to less chronic pain intensity and pain interference in addition to better performance of daily activities [58,59]. Preliminary research has also found that trait optimism is associated with lower levels of pain intensity and disability in individuals with chronic pain [30]. Previous observational studies have shown that pain self-efficacy partially mediates the longitudinal association between changes in pain and changes in disability in individuals with chronic low back pain [37], as well as pain self-efficacy and pain acceptance cross-sectionally mediate the association between pain intensity and engagement in valued activities in people with rheumatoid arthritis [35]. Several studies have found that self-efficacy beliefs [38] and pain acceptance [39,40,41,42] mediate the improvement in psychological and multidisciplinary pain treatments in individuals with chronic pain.

The results of our study are not only consistent with these findings supporting the role that positive psychological factors play in the adjustment to chronic pain but contribute new important knowledge regarding the nature of this role. All these results are in agreement with theoretical pain models that underline the importance of positive psychological factors in the experience of chronic pain. For example, Sturgeon and Zautra proposed that positive cognitive factors (i.e., acceptance), as well as positive affective states (i.e., happiness), foster the use of active coping responses that drive chronic pain patients towards a better resilience response (adaptation) to that pain [17]. This model has been also tested by previous research that showed the importance of resilience factors in the adjustment of chronic pain in adult and pediatric populations [60,61]. Additionally, the psychological flexibility model also empathizes the importance of resilient factors such as pain acceptance in the management of chronic pain symptoms [18]. Previous research has found how different factors included in this model such as pain acceptance and cognitive defusion can be relevant in the improvement of biopsychosocial domains (social and emotional) in people with chronic pain [62]. This model is also the core of Acceptance and Commitment Therapy which has been shown to reduce chronic pain symptoms such as anxiety, depression, and disability [63].

To our knowledge, this is the first study that evaluates multiple positive psychological factors in the same sample and analysis. We found that all three positive psychological factors contributed significantly to the prediction of pain interference, even when controlling for age, gender, pain intensity, and the other two factors. Moreover, we found that both pain self-efficacy and pain acceptance, but not trait optimism, had significant—and similar—cross-sectional mediating effects in the association between pain intensity and pain interference. On the other hand, the multiple mediation model did not obtain a significant effect and none of the three positive psychological factors cross-sectionally moderated the association between pain intensity and pain interference.

The lack of cross-sectional mediation or moderation effects for optimism as well as the direction of the significant direct effects on pain interference was not anticipated, given previous findings showing that greater optimism is associated with less pain and disability in a large sample of individuals with chronic musculoskeletal pain [30]. One factor that may account for the inconsistency between our results and those from the previous study is the presence of a larger sample and stronger statistical power compared to our study. Therefore, our effects may be real, but weak. If these findings of weak and/or not-significant effects of optimism are replicated in other samples, then these suggest that trait optimism may have a limited role in helping individuals adjust to chronic musculoskeletal pain. Treatments that focus on variables that may play a more important role in the outcome, such as pain self-efficacy or pain acceptance, may ultimately be more helpful. Research is needed to evaluate this possibility.

Another factor that could explain the relative weakness of the effects associated with optimism is that the measure of optimism used assesses general optimism about life, and not optimism associated with pain, specifically. The other two positive psychological factors examined in this study were both pain-specific. Thus, a general measure of self-efficacy (rather than pain self-efficacy) may have also evidenced weak and non-significant effects concerning predicting pain interference. Perhaps a pain-specific measure of optimism (e.g., assessing hope in a future in which pain may be present but manageable) might have evidenced stronger associations. A measure assessing pain-related optimism would need to be developed to test this idea.

The positive (and similar) findings concerning pain self-efficacy and pain acceptance as cross-sectional mediators but not moderators of the association between pain intensity and pain interference have important clinical implications. First, despite the multiple mediation model not presenting a significant effect, when both factors were separately introduced this showed significant effects that may suggest that treatments should not necessarily focus on one mechanism variable over the other – treatments that seek to alter both might be expected to be more effective than those that target only one. Second, the lack of cross-sectional moderator effects suggests that it is not so important how much pain self-efficacy and pain acceptance one has in general (i.e., before a change in pain), but how able an individual is to elicit high levels of pain self-efficacy and pain acceptance in response to increase in pain intensity that is most important. Thus, the findings suggest that treatments which help patients who already have chronic pain respond to increase in pain intensity by [i] focusing on what they are capable of doing to manage the pain (i.e., pain self-efficacy) and [ii] being better able to accept the pain as pain waxes and wanes might be particularly helpful. However, these hypotheses should not be confirmed with the present cross-sectional data. The results obtained in the present study must be tested in longitudinal studies before drawing any causal conclusion.

Importantly, we found that both pain self-efficacy and pain acceptance only partially and cross-sectionally mediated the association between pain intensity and pain interference. Thus, additional factors likely play a role. For example, one cross-sectional study has shown that a positive psychological factor—psychological flexibility—mediates the association between symptom severity (both pain intensity and anxiety) and function (i.e., pain interference and depression) in a sample of individuals with chronic pain [64]. Another cross-sectional study also showed how psychological vulnerability factors such as catastrophizing, depression, and fear mediate the association between pain and disability in individuals with chronic low back pain [65]. One study found that a psychological vulnerability factor—psychological inflexibility—mediated the effects of Acceptance and Commitment Therapy on pain interference and life satisfaction in a sample of individuals with chronic pain following whiplash [66]. Thus, additional research is needed to evaluate a larger number of both positive and negative psychological factors in the same sample to determine which plays the most important roles and determine if as a group these factors can explain more mediation effects than single or subsets of these variables. According to this point, two recent studies in people with fibromyalgia have shown how high resilience and low pain catastrophizing reduced fibromyalgia severity via low distress [67] and how high cognitive fusion and high pain catastrophizing increased functional limitations via fibromyalgia symptoms (depression, pain, and physical fatigue) [68].

### Limitations

The study has a number of limitations that should be considered when interpreting the findings. First, we used a cross-sectional design to evaluate potential mediation effects. As such, it represents a correlational study, which does not allow for any conclusions regarding the possible causal impact of the mediation variables studied and function. Future longitudinal and experimental (e.g., randomized clinical trials) studies in which the mediational variables are systematically manipulated (e.g., using positive psychology interventions versus control conditions) and the effects of these changes on function are evaluated. Additionally, all of the study measures were self-report measures. As a result, some of the significant associations identified could be due to shared method variance or social desirability bias. Future researchers should consider using observational measures (e.g., of physical activity or sleep, such as actigraphy [69]) when possible, to reduce this potential source of bias. Finally, the sample consisted of patients with chronic musculoskeletal pain who were seen in specialty pain clinics. It is not possible to determine the extent to which the findings from this sample generalize to individuals with other chronic pain conditions (e.g., headache, abdominal pain, or neuropathic pain), patients from other clinical settings, or individuals with chronic pain in the community. Research using individuals from these other populations or in additional settings is needed to evaluate the generalizability of the current findings.

## 5. Conclusions

Despite the study’s limitations, the findings provide important new information regarding the role that positive psychological factors may play in the adjustment to chronic pain. This descriptive study using mediation analyses found that both pain self-efficacy and pain acceptance, but not trait optimism, partially and cross-sectionally mediated the association between pain intensity and pain interference. Also, these cross-sectional mediation effects were weak, indicating that other factors—perhaps other positive/negative cognitive domains as well as positive/negative emotional domains (i.e., emotional intelligence) [70]—also play additional unique roles in the adjustment to chronic pain. Furthermore, the multiple mediation model did not obtain a significant effect. The lack of cross-sectional moderation effects, if replicated in other studies, suggests the possibility that for individuals to benefit most from these factors, clinicians should focus on teaching individuals to elicit pain self-efficacy beliefs and pain acceptance in response to increase in pain specifically, rather than on being accepting (of pain) or having more self-efficacy beliefs in general. Future research in other samples, using objective measures (when possible), and using longitudinal and experimental designs are needed to test these ideas and evaluate the reliability and generalizability of the study findings.

## Figures and Tables

**Table 1:** Baseline demographic and clinical characteristics of the study sample.

Variable	Mean (SD) or Number (%)
Age in years (*n* = 186)	52.7 (10.6)
Gender (*n* = 186)	
Female	133 (71.5%)
Male	53 (28.5%)
Duration of symptoms (*n* = 186)	
2–6 months	12 (6.5%)
6–12 months	12 (6.5%)
>12 months	162 (87.1%)
Pain site (*n* = 183)	
Low back	78 (42.6%)
Neck	41 (22.4%)
Shoulder	43 (23.5%)
Knee/hip	11 (6.0%)
Other sites	10 (5.5%)
Employment status (*n* = 184)	
Employed	73 (39.7%)
Unemployed	28 (15.2%)
On sick leave	14 (7.6%)
Retired	30 (16.3%)
Homemaker	39 (21.2%)
Educational level (*n* = 184)	
No formal education	8 (4.3%)
Primary school	50 (27.2%)
Secondary school	72 (39.1%)
Bachelor’s degree,	47 (25.5%)
Masters and/or PhD	7 (3.8%)
Primary treatment received (*n* = 186)	
No treatment	41 (22.0%)
Pharmacological treatment	18 (9.7%)
Physiotherapy	117 (62.9%)
Other treatment (e.g., acupuncture)	8 (4.3%)
Presence of comorbidities (*n* = 186)	
Yes	103 (55.5%)
No	81 (43.5%)
Pain intensity (GCPS pain subscale score: 0–30, *n* = 161)	17.3 (SD, 6.7) [min 0 to max 30]
Pain interference (GCPS pain interference subscale score: 0–30 *n* = 160)	17.5 (SD, 12.1) [min 0 to max 40]
Pain self-efficacy (PSEQ score: 0–60, *n* = 180)	37.6 (SD, 14.6) [min 2 to max 60]
Optimism (LOT-R score: 0–40, *n* = 160)	15.8 (SD, 4.2) [min 4 to max 24]
Pain acceptance (CPAQ total score: 0–120, *n*= 153)	62.6 (SD, 19.2) [min 15 to max 114]

Note SD = standard deviation; GCPS = Grade Chronic Pain Scale; PSEQ = Pain Self-Efficacy Questionnaire; LOT-R = Life Orientation Test-Revised; CPAQ = Chronic Pain Acceptance Questionnaire.

**Table 2:** Correlation coefficients between psychological measures, pain intensity, and pain interference.

	Pain Intensity (GCPS)	Pain Interference (GCPS)	Pain Self-Efficacy (PSEQ)	Optimism (LOT-R)
Pain interference (GCPS)	0.71 *	-	-	-
Pain self-efficacy (PSEQ)	−0.44 *	−0.50 *	-	-
Optimism (LOT-R)	−0.13	−0.08	0.28 *	-
Pain acceptance (CPAQ)	−0.40 *	−0.51 *	0.54 *	0.41 *

Note GCPS = Grade Chronic Pain Scale; PSEQ = Pain Self-Efficacy Questionnaire; LOT-R = Life Orientation Test-Revised; CPAQ = Chronic Pain Acceptance Questionnaire; * *p*-value significant at 0.01.

**Table 3:** Results of the linear regression analyses model predicting pain interference from pain intensity, pain self-efficacy, pain acceptance, and optimism.

				95% CI			
Model (R^2^)		β	*p*	LCI	UCI	Tolerance	VIF
1 (0.50)	(Constant)		<0.001	8.99	23.47		
	Pain intensity	0.71	<0.001	1.06	1.48		
2 (0.59)	(Constant)		<0.001	10.17	23.56		
	Pain intensity	0.56	<0.001	0.78	1.21	0.75	1.32
	Pain self-efficacy	−0.14	0.026	−0.23	−0.01	0.64	1.56
	Pain acceptance	−0.28	<0.001	−0.26	−0.08	0.59	1.67
	Optimism	0.14	0.015	0.07	0.76	0.82	1.22
3 (0.60)	Pain intensity * Pain Self-Efficacy	−0.04	0.538	−0.02	0.01	0.64	1.15
	Pain intensity * Pain Acceptance	−0.06	0.407	−0.02	0.01	0.56	1.78
	Pain intensity * Optimism	0.01	0.825	−0.04	0.06	0.74	1.35

Durbin–Watson = 2.02; CI = confidence interval; VIF = variance inflation factor; LCI = lower confidence interval; UCI = upper confidence interval. The three models were adjusted by age and gender. * Interaction between pain intensity and each psychological factor (moderator effects of each psychological factor in the association between pain intensity and pain interference).
